# Corrigendum to “Protective Effects of Pretreatment with Oleanolic Acid in Rats in the Acute Phase of Hepatic Ischemia-Reperfusion Injury: Role of the PI3K/Akt Pathway”

**DOI:** 10.1155/2020/9649787

**Published:** 2020-11-28

**Authors:** Bo Gui, Fuzhou Hua, Jie Chen, Zeping Xu, Hongbin Sun, Yanning Qian

**Affiliations:** ^1^Department of Anesthesiology and Perioperative Medicine, 1st Affiliated Hospital, Nanjing Medical University, Nanjing, China; ^2^Key Laboratory of Anesthesiology, Xuzhou, Jiangsu, China; ^3^Department of Anesthesiology, 2nd Affiliated Hospital, Nanchang University, Nanchang, China; ^4^Department of Emergency, 1st Affiliated Hospital, Nanjing Medical University, Nanjing, China; ^5^Department of Anesthesiology, Jiangsu Tumor Hospital, Nanjing, China; ^6^Center for Drug Discovery, College of Pharmacy, China Pharmaceutical University, Nanjing, China

In the article titled “Protective Effects of Pretreatment with Oleanolic Acid in Rats in the Acute Phase of Hepatic Ischemia-Reperfusion Injury: Role of the PI3K/Akt Pathway” [1], there was an error in Figure 6. The incorrect images were presented as the representative images for the p-GSK-3*β* bands at the preoperative (Prep) stage and their corresponding GSK-3*β* bands in Figure 6.

The corrected figure is shown below and is listed as Figure 1.

## Figures and Tables

**Figure 1:**
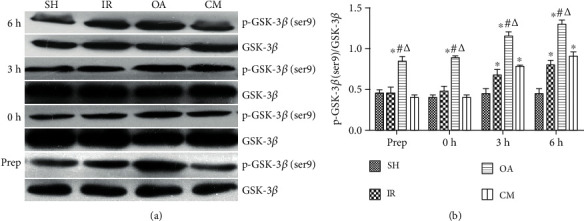
Effects of pretreatment with OA on p-GSK-3*β* (ser9) and GSK-3*β* protein expression in rats induced by partial hepatic ischemia-reperfusion (IR). Expression of p-GSK-3*β* (ser9) and GSK-3*β* protein was detected by western blot analysis (a). These bands were quantified and analyzed (b). Data are represented as mean ± SD (*n* = 8). ∗*P* < 0.05, compared with group SH. ^#^*P* < 0.05, compared with group IR. *^Δ^P* < 0.05, compared with group CM.
